# Special Issue: “New Trends in Diabetes, Hypertension, and Cardiovascular Diseases”

**DOI:** 10.3390/ijms25052711

**Published:** 2024-02-27

**Authors:** Yutang Wang, Dianna J. Magliano

**Affiliations:** 1 Discipline of Life Science, Institute of Innovation, Science and Sustainability, Federation University Australia, Ballarat, VIC 3350, Australia; 2 Diabetes and Population Health, Baker Heart and Diabetes Institute, Melbourne, VIC 3004, Australia

Cardiovascular disease (CVD) encompasses a range of disorders affecting the heart and blood vessels, including coronary heart disease and cerebrovascular disease [1] and conditions such as aortic aneurysms and lower-extremity peripheral artery disease [2]. It remains the leading cause of death worldwide, claiming 17.9 million lives annually [3], despite the availability of numerous therapeutic drugs [4]. Thus, further research is warranted to enhance CVD treatment and reduce its morbidity and mortality [5].

Diabetes is a leading cause of blindness, kidney failure, heart attacks, stroke, and lower-limb amputation, causing an estimated 2 million deaths annually [6]. In 2021, 529 million individuals (prevalence, 6.1%) were afflicted with diabetes, a figure that is projected to soar to 1.21 billion by 2050 [7]. Diabetes represents a significant public health concern worldwide [8]. Despite remarkable strides in prevention and treatment, further research is required. This is particularly important given the recent introduction of new efficacious medications and the developments in AI technology, the latter being useful in the development of precision medicine algorithms to treat people with diabetes [9].

Hypertension impacts 1.28 billion individuals aged 30–79 worldwide [10], serving as a critical risk factor for ischemic heart disease, stroke, chronic kidney disease, and dementia, with approximately 10 million deaths attributed to it annually [11]. The number of people suffering from hypertension doubled from 1990 to 2019 [12], with only 22.5% of hypertensive adults having their condition under control [13]. Hence, improved detection, treatment, and control strategies are imperative.

Diabetes and hypertension are conditions commonly found in aging populations; they share common pathogenic pathways [14] and are both strong independent risk factors for CVD. Reducing blood glucose and blood pressure levels can significantly contribute to mitigating CVD-related morbidity and mortality.

This Special Issue aims to present the latest perspectives and research findings on diabetes, hypertension, and CVD, comprising eight review articles and eight original research articles.

The first review, “The Role of the Gut Microbiome and Trimethylamine Oxide in Atherosclerosis and Age-Related Disease”, explores the contribution of the gut microbiome and trimethylamine oxide to CVD, emphasizing the potential utility of reducing trimethylamine oxide as a novel therapeutic strategy.

The second review, “The Role of Oxidative Stress Enhanced by Adiposity in Cardiometabolic Diseases”, delves into the contribution of adiposity-associated oxidative stress to cardiometabolic diseases, offering insights into the mechanisms underlying this interplay.

The third review, “Portrayal of NLRP3 Inflammasome in Atherosclerosis: Current Knowledge and Therapeutic Targets”, examines the role of the NLRP3 inflammasome in atherosclerosis, highlighting its potential as a therapeutic target.

It is followed by “A Receptor Story: Insulin Resistance Pathophysiology and Physiological Insulin Resensitization’s Role as a Treatment Modality”, which discusses the pathophysiology of insulin resistance, emphasizing the therapeutic potential of dynamic exogenous insulin administration.

Next, “Smooth Muscle Heterogeneity and Plasticity in Health and Aortic Aneurysmal Disease” explores vascular smooth muscle cell phenotypes in aortic aneurysms, calling for further research into their relationship with aneurysm formation.

The sixth review, “Angiotensin Regulation of Vascular Homeostasis: Exploring the Role of ROS and RAS Blockers”, highlights the role of the renin–angiotensin system (RAS) in CVD pathogenesis and the potential of RAS blockers to reduce vascular oxidative stress.

Next, “Dysfunctional and Dysregulated Nitric Oxide Synthases in Cardiovascular Disease: Mechanisms and Therapeutic Potential” examines the contribution of nitric oxide synthases to cardiovascular health and their potential as therapeutic targets.

Finally, “Model Systems to Study the Mechanism of Vascular Aging” explores various models of vascular aging, providing insights into the aging process.

The original research articles delve into various aspects of CVD, shedding light on sex-specific features, genetic associations, and the effects of lifestyle factors, such as night shifts, on disease risk.

The first, “Sex-Specific Features of the Correlation between GWAS-Noticeable Polymorphisms and Hypertension in Europeans in Russia”, examines the sex-specific features of genome-wide-association-studies-identified single-nucleotide polymorphisms (SNPs) in relation to hypertension. This study revealed that hypertension-associated SNPs had a more pronounced effect on susceptibility in women than in men.

The second research paper, “SERPINE1 mRNA Binding Protein 1 Is Associated with Ischemic Stroke Risk: A Comprehensive Molecular–Genetic and Bioinformatics Analysis of SERBP1 SNPs”, investigated the association between genetic variants of serpin family E member 1 (SERBP1) and ischemic stroke risk. The findings suggest that SERBP1 SNPs may serve as novel genetic markers for stroke.

The third, “Preliminary Study on the Effect of a Night Shift on Blood Pressure and Clock Gene Expression”, examined the effect of night shifts on ambulatory blood pressure and clock gene expression. The study revealed that night shifts negatively impacted ambulatory blood pressure, leading to a non-dipping blood pressure status, which is a risk factor for CVD. Moreover, night shifts were associated with an increase in clock gene expression.

The fourth research paper, “Age-Dependent Changes in the Relationships between Traits Associated with the Pathogenesis of Stress-Sensitive Hypertension in ISIAH Rats”, investigated the impact of age on the development of stress-sensitive hypertension. The study identified age as a factor affecting hypertension manifestation.

The fifth research study, “Prognostic Significance of Activated Monocytes in Patients with ST-Elevation Myocardial Infarction”, examined the prognostic efficacy of monocyte subsets in patients with ST-elevation myocardial infarction. The study found that elevated levels of nonclassical (CD14^+^CD16^++^) monocytes and their subsets predicted worse clinical outcomes in these patients.

Next, “Augmentation of Cathepsin Isoforms in Diabetic db/db Mouse Kidneys Is Associated with an Increase in Renal MARCKS Expression and Proteolysis” investigated the expression and proteolysis of key proteins in the kidneys of diabetic db/db mice. The study revealed a significant cleavage of the myristoylated alanine-rich C-kinase substrate (MARCKS) family of proteins, important in maintaining normal renal function, in diabetic mice. This increase in MARCKS cleavage was associated with an increase in the protein expression of cathepsin isoforms.

Following this, “Tumor Growth Ameliorates Cardiac Dysfunction and Suppresses Fibrosis in a Mouse Model for Duchenne Muscular Dystrophy” explored the association between heart failure and cancer in mice. The study found that tumour growth ameliorated cardiac dysfunction and reduced overall fibrosis, suggesting potential novel strategies to improve cardiac function and fibrosis-associated diseases by understanding tumour paradigms.

Finally, “Effect of Hydralazine on Angiotensin II-Induced Abdominal Aortic Aneurysm in Apolipoprotein E-Deficient Mice” investigated the impact of hydralazine on abdominal aortic aneurysms in a mouse model. The study concluded that hydralazine inhibited the formation and rupture of abdominal aortic aneurysms, attributed to its anti-inflammatory and anti-apoptotic properties.

In conclusion, this Special Issue provides a comprehensive overview of recent developments in research on hypertension, insulin resistance/diabetes, and CVD, including atherosclerosis, aortic aneurysms, and myocardial infarction [15]. These articles offer insights into CVD pathogenesis and facilitate the development of new therapeutic approaches.
